# Alteration in HDEMG Spatial Parameters of Trunk Muscle Due to Handle Design during Pushing

**DOI:** 10.3390/s21196646

**Published:** 2021-10-06

**Authors:** Jacqueline Toner, Jeremy Rickards, Kenneth Seaman, Usha Kuruganti

**Affiliations:** 1 Andrew and Marjorie McCain Human Performance Laboratory, Faculty of Kinesiology, University of New Brunswick, Fredericton, NB E3B5A3, Canada; jc526075@dal.ca; 2 Faculty of Forestry and Environmental Management, University of New Brunswick, Fredericton, NB E3B5A3, Canada; jeremyrickards6@gmail.com; 3 Faculty of Kinesiology, University of New Brunswick, Fredericton, NB E3B5A3, Canada; kseaman@unb.ca

**Keywords:** industrial cart, handle design, trunk muscles, high-density electromyography

## Abstract

Previous research identifies that pushing and pulling is responsible for approximately 9–18% of all low back injuries. Additionally, the handle design of a cart being pushed can dramatically alter a worker’s capacity to push (≅9.5%). Surprisingly little research has examined muscle activation of the low back and its role in muscle function. Therefore, the purpose of this study was to examine the effects of handle design combination of pushing a platform truck cart on trunk muscle activity. Twenty participants (10 males and 10 females, mean age = 24.3 ± 4.3 years) pushed 475 lbs using six different handle combinations involving handle orientation (vertical/horizontal/semi-pronated) and handle height (hip/shoulder). Multichannel high-density EMG (HDsEMG) was recorded for left and right rectus abdominis, erector spinae, and external obliques. Pushing at hip height with a horizontal handle orientation design (HH) resulted in significantly less (*p* < 0.05) muscle activity compared to the majority of other handle designs, as well as a significantly higher entropy than the shoulder handle height involving either the semi-pronated (*p* = 0.023) or vertical handle orientation (*p* = 0.028). The current research suggests that the combination of a hip height and horizontal orientation handle design may require increased muscle demand of the trunk and alter the overall muscle heterogeneity and pattern of the muscle activity.

## 1. Introduction

Workplace ergonomics aims to systematically reduce risk factors to protect workers and prevent costly injuries, while simultaneously providing improvement for an employer in terms of increased productivity and lower costs associated with injuries [1]. By developing ergonomic strategies, employers can save money by avoiding both the direct and indirect cost of an injury due to the reduced risk of injury. In the manufacturing industry, pushing and pulling tasks increase the risk of low back injury and are considered a major contributor to overall injury [2]. Often, pushing and pulling tasks are completed using an industrial cart with limited adjustability and little design consideration regarding worker anthropometrics. However, it has been shown that cart design can impact a worker’s overall capacity to push by as much as 9.5% [3]. Given the extensive use of industrial carts in workplaces across several industries, there is surprisingly little research regarding the physical demands of workers pushing carts. Surface electromyography (EMG) is widely used to measure muscle load in ergonomics. The surface EMG signal can be used to provide valuable information regarding muscle timing as well as relative intensity of muscles during work, which can be used to determine job-related task requirements and limitations [4]. Furthermore, surface EMG is noninvasive, requires minimal training, and can be performed wirelessly, making it well suited for use during typical work operations without worker interference, particularly when integrated with other measures of physical workload such as heart rate and force. More specifically, surface EMG has been used to examine muscle activity during pushing tasks [5,6,7] and has been shown to be useful to identify physiologic conditions impacting worker health such as muscle fatigue, activity, and recruitment. For example, pushing tasks require nearly twice the muscle activation, depending on the height of the handles [5], compared to pulling. This results in approximately twice the EMG amplitude, suggesting an increased muscle load. It has also been shown that lower muscle activity and trunk stiffness were identified when using a shoulder-height cart design compared to a hip-height design, due to higher trunk inclination after the onset of the task [2]. With respect to the lower back, previous researchers found that both pushing and pulling loads equivalent to 20% of a workers’ body weight appeared to be the limit of acceptable exertions for their upper lumbar spine [8]. While improper handle design may negatively affect workers during cart use [2,5], research examining handle design during wood routing tasks found no significant differences in the EMG from the biceps brachii and extensor carpi radialis brevis using seven different designs [9]. While limited, these previous studies suggest a greater understanding of muscle load during pushing, and changes due to handle design may provide useful design information for ergonomists and design engineers.

Changes found among sEMG signals are related to the continuous modifications in force output, muscle fiber length, and relative position of surface electrodes and sources [10]. Because of these variables, engineers have aimed to further improve the reliability of the information extracted from sEMG using multichannel recordings to overcome some limits of the standard technique [10]. Multichannel recordings (i.e., those using multiple electrodes) have become the gold standard for noninvasive examination of muscle activation patterns [11,12]. These larger arrays of electrodes, also known as high-density EMG (HDEMG), involve electrodes arranged on a two-dimensional grid, which is placed over a muscle belly. The amplitude of the individual signals allows for the development of a topographical or spatial map representing muscle electrical activity over the skin plane [13,14]. HDEMG provides information regarding the spatial distribution of electric potential over the skin surface during muscle contraction [15], and features of the spatial map can be used to provide insight into muscle activation.

Furthermore, the higher discriminative power offered through HDEMG data offers an improved methodology among certain tasks and extends the possibilities offered by currently available instrumental-based tools [16]. Previously, it has been shown that the spatial activation distribution in a muscle is nonuniform and that the EMG spatial distribution pattern can be altered by contraction levels or fatigue [11,15,17]. Features of the distribution map can also be used to assess muscle function. For example, it has been shown that motor unit (MU) loss and changes in muscle fiber composition can result in “clustering” or large areas of muscle being occupied by similar types of muscle fibers [17,18,19,20]. The clustering of fibers has been suggested to indicate low heterogeneity of muscle fibers [20]. Features such as entropy of the electrical signal and the coefficient of variation (CoV) have been used to characterize the heterogeneity in the spatial multichannel HDEMG potential distribution [15,20,21]. Additionally, it has been established that the muscle intensity and spatial distribution of HDEMG maps could be useful in applications where the identification of movement intention and its strength is needed [13], making this technology particularly useful for assessing workplace tasks such as pushing and pulling.

It is estimated that approximately 9–18% of all low back injuries are associated with pushing and pulling tasks [22,23,24,25,26,27,28,29]. While the activity of pushing and pulling can contribute to low back injuries in workers, the design of the cart has not changed significantly over the years despite the understanding that inappropriate design and/or use can lead to worker injuries. Furthermore, while most industrial carts in use have some adjustability, the design could be improved by increasing our understanding of the impact of features such as handle design on muscle load. For example, researchers found that workers pushing at shoulder height may be at a higher risk of a low back injury than when they were pushing at their hip height [2]. In addition, it has been shown that semi-pronated and horizontal handle orientations produced greater pushing force than the vertically oriented handles [3]. An earlier study found that lower forces were applied during whole-body pushing task exertions when using horizontal handles, which, in turn, limited the amount of muscle recruitment needed for the task [30]. These variations of applied pushing force may contribute to low back concerns and obtaining information regarding trunk muscle activity using HDEMG may provide insight regarding muscle function and compensation during pushing tasks.

To our knowledge, there are no studies that have examined spatial muscle activity during pushing and pulling with different handle configurations. HDEMG may provide greater information regarding the spatial recruitment of motor units within a muscle [31], and spatial features may provide valuable information regarding muscle function during pushing tasks. Therefore, the purpose of this study was to use HDEMG to examine the effects of handle design on spatial features of trunk muscle activity during a simulated industrial cart-pushing task. The results obtained will help to improve handle design and develop improved work protocols.

## 2. Materials and Methods

### 2.1. Participants

Twenty individuals (10 males and 10 females) between 19 and 60 years of age participated in this study. This sample size was determined based on previous literature and a power calculation indicating a minimum of eight participants of each sex. The general characteristics of the participants are provided in Table 1. Exclusion criteria included: any history or symptoms of a fracture or dislocation of the spine, a torn or ruptured disc, cauda equina syndrome, having a fused spine, scoliosis/kyphosis/lordosis, and/or spondylosis. Furthermore, skin-fold testing of the right rectus abdominis and obliques location needed to be lower than 40 mm, as those with higher levels of adipose tissue in these specific areas could interfere with the HDEMG recordings. Participants were provided an overview of all testing protocols and associated risks and asked to sign an informed consent form. All testing was conducted at the University of New Brunswick and was approved by the Research Ethics Board (REB) before the commencement of any data collection (REB 2019-060).

### 2.2. Instrumentation

A 64-channel wireless (Sessantaquattro, OT Bioelettronica, Turin, Italy) HDEMG system was used to record all muscle activity. The Sessantaquattro is a multichannel amplifier and data logger for bioelectrical signals. The signals acquired by the instrument were amplified, filtered, digitally converted, and transferred to a PC, through a WiFi connection, for real-time visualization and storage. The monopolar EMG signals were sampled at 1000 Hz, with a low-pass filter of 500 Hz and a high-pass filter of 10 Hz. The EMG signals were captured with two semi-disposable 32-channel electrode grids (grid ID = ELSCH4 × 8NM6). A ground electrode was placed on the right ankle to avoid signal interference by the participant’s heart rate. The HDEMG data were used to create heat intensity maps of the three trunk muscles when performing the dynamic task of pushing with the various handle setups. Spatial features were then extracted from the heat intensity maps for further analysis.

### 2.3. Surface EMG Preparation

Standard skin preparation techniques were used for this research study to ensure proper electrode–skin contact [32]. All muscle sites were shaved using shaving gel at the location of electrode placement to improve the electrode adhesion to the skin. The skin was then cleaned with alcohol swabs to remove any dirt, oil, and sweat. Following the use of the alcohol swab, rough paper-towel was brushed over the skin to remove any debris or loose skin cells that may impede the electrical signal. The participant’s skin was prepared using abrasive paste (Meditec-Every, Parma, Italy) and water, and two adhesive grids, each consisting of 32 electrodes (LISiN-OT Bioelettronica, Torino, Italy, model ELSCH4x8NM6), were placed, by the same researcher, over the left and right muscle bellies of either the rectus abdominis, external obliques, or the erector spinae. Grids were placed on the participant at the designated muscle site and secured in place using Hypafix (BSN Medical, Hamburg, Germany) to prevent the electrode from detaching from the skin. While all grids were placed on the muscles of interest at the same time, only 64 channels could be collected at one instance due to the limitation of the EMG system. Therefore, the participant was asked to complete a total of 18 trials to ensure that sufficient data were collected for the three trunk muscles and six handle designs.

**Figure 1:**
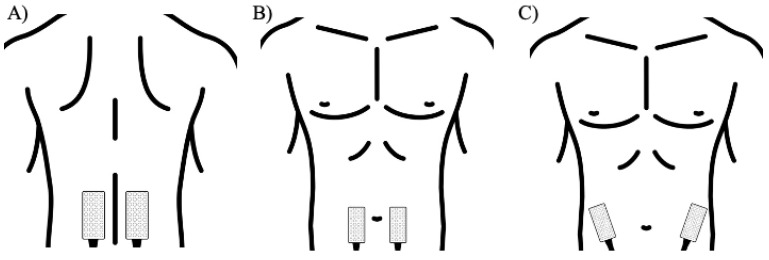
Illustrated HDEMG grid placement of the (**A**) erector spinae, (**B**) rectus abdominis (**C**) external obliques.

Each grid consisted of eight rows and four columns of electrodes (1 mm diameter, 8 mm interelectrode distance in both directions), with one electrode absent in the upper right corner. All of the electrode placements were executed according to validated protocols [33]. Electrode placement was done such that the columns of HD electrodes were oriented parallel to the muscle fibers of the underlying muscle. For the rectus abdominis, the HD grid was located approximately 2 cm lateral and across from the umbilicus over the muscle belly; for the external oblique, electrodes were placed lateral to the rectus abdominis and directly above the anterior superior iliac spine, halfway between the crest and the ribs at a slightly oblique angle; and lastly, for the erector spinae, electrodes were placed one finger width medial from the line from the posterior superior iliac spine to the lowest point of the lower rib, at the level of L2. Electrode grid placements are shown in Figure 1.

### 2.4. Study Design

**Figure 2:**
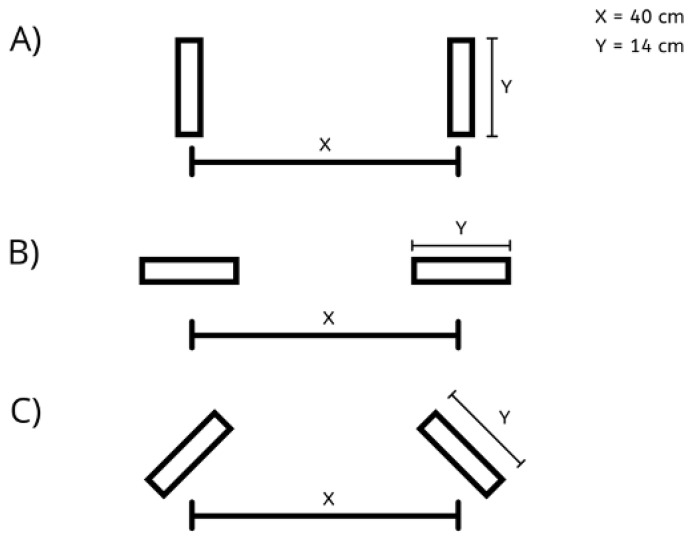
Handle orientations: (**A**) vertical, (**B**) horizontal, and (**C**) semi-pronated included in the handle designs. Interhandle distance (X) between the two handles was a distance of 40.0 cm, and the length of the handle (Y) itself was 14.0 cm.

Participants were required to push a platform cart in a straight line over five meters to minimize the impacts of tiredness insinuated by the task itself and limit the push to the initiation phase. The cart was loaded with 216 kg and was instrumented with six varying handle designs: vertical (V), horizontal (H), and semi-pronated (S) (Figure 2) at two different heights: shoulder (S) and hip (H). The weight of 216 kg was selected based on the available weights and previous handle design research [2]. In addition, the weight was the same for both sexes as this is typical industrial practice.

**Figure 3:**
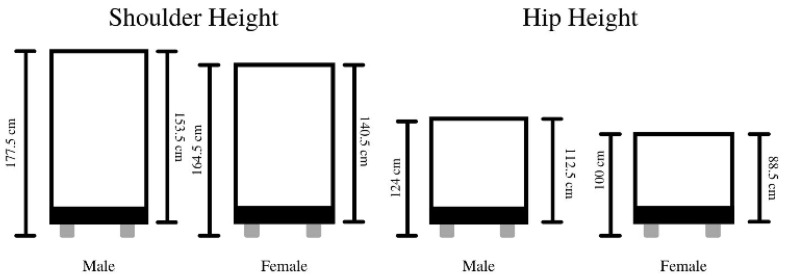
Illustration of the two handle heights for both males and females.

The handle heights used within this study were altered from the original cart, as this allowed for differences in both male and female average anthropometric values. The average shoulder and hip height of males (shoulder: 153.5 cm, hip: 100.0 cm) and females (shoulder: 140.5 cm, hip: 88.5 cm) [34] were used to ensure the most accurate results between the individual sex anthropometrics. Diagrams displaying the two handle heights of males and females can be found in Figure 3.

Participants were asked to complete a total of six trials: one trial for each possible combination of the three-handle orientations and two handle-height configurations specific to their sex. The order of the trials was randomized. Prior to the start of each trial, each participant was instructed on proper form and their starting position. Specifically, participants were instructed to stand such that their dominant leg was located approximately 5 cm behind their non-dominant leg, their feet were to be placed shoulder-width apart, and they were instructed to stand centered in front of the test handles. Participants were also instructed to have their arms outstretched, gripping onto the handles with a slight bend in their elbow, while also looking straight ahead with their chest lifted. If, at any point, the researcher deemed that the participant’s posture was incorrect, then the trial was retested. To further prevent bias in participant technique, the same researcher was present for all testing. Errors in a participant’s posture included having overly outstretched arms (locked elbows), an extreme forward or backwards tilted pelvis, narrow foot placement, and having one’s chest aligned with their wrists. The speed of each push was a free choice of the participant, and they were instructed to move at their typical walking pace. Once the participant was ready to start the trial, the researcher conducted a countdown of “three, two, one, and go”, followed by the participant completing their pushing trial. All systems started recording three seconds before the countdown to ensure that a quiet signal was recorded as a baseline for both force and HDEMG recordings.

### 2.5. Data Analysis

All EMG data were filtered with a bandpass filter set at 10–500 Hz and were then exported and processed offline using custom software (Matlab; Mathworks Inc., Natick, MA, USA). The EMG amplitude of each signal was normalized to the individual’s maximum isometric voluntary contraction (MVC) for calibration and comparison. The MVC was obtained by having the participant complete three five-second maximal contractions of each muscle of interest (erector spinae, obliques, and rectus abdominis).

The recorded signals were analyzed offline using the custom-built open-source MATLAB software titled HDsEMG Analysis Tool (Version 1.0; https://github.com/ashirbadpradhan/HDsEMG-Analysis-Tool-1.0 accessed on 1 November 2019). The following variables were computed to compare the group data: intensity, differential intensity, mean RMS, modified entropy, and coefficient of variation.

It has been suggested that the relationship between EMG amplitude and force generated is not linear [35,36], and therefore intensity was defined, similarly to previous work [11,12,37,38], as the common logarithm of the mean intensity of the HDEMG maps:

$$I=\log_{10}\frac{1}{N}\sum_{i,j}{\mathrm{HM}}_{i,j}$$

where I is an intensity feature calculated from the HDEMG intensity map HM with a total number of N channels, and HM_i,j_ is the intensity of a channel located at position i,j. The differential intensity (DI), which represents the intensity of a single differential channel, was calculated as a common logarithm of an RMS value of the difference of two consecutive channels in the direction of the muscle fibers [12]:

$$\mathrm{DI}=log_{10}\left(\mathrm{RMS}\left(sEMG_{i,j}-sEMG_{i+1,j}\right)\right)$$

Distribution of muscle activity was estimated using the RMS value for each of the electrode grid locations for each participant, and two-dimensional (2D) maps were developed for each participant. The HDEMG maps represent the spatial distribution of intensities of active MUs over the surface of the muscle, as follows [12,37,38]:

$$HM_{ij}=\mathrm{RMS}(sEMG_{ij})$$

where HM is an activation map and each pixel in a map (HM_i,j_) corresponds to an RMS value of a channel in an electrode array (position i,j).

The heterogeneity of the spatial activity was characterized with modified entropy and coefficient of variation. Maximum entropy occurs when all the channels have the same RMS (log_2_28 = 4.8), and, conversely, minimum value occurs when all of the channels are 0 except for one. Therefore, an increase in entropy indicates an increase in homogeneity of muscle fiber types, since muscle fibers innervated by the same motor neuron will have similar EMG recordings. Modified entropy was defined as the entropy of the signal power [16,37,38]:

$$E=-\sum_{i=1}^{28}p{\left(i\right)}^{2}log_{2}p{\left(i\right)}^{2}$$

where p(i)^2^ is the square of the RMS value of channel i divided by the sum of the squares of all 32 RMS values. Thus, p(i)^2^ denotes the normalized power of each channel.

The CV was calculated as the standard deviation (SD) of the 32 RMS values divided by the average of the 32 RMS values.

When the SD is small relative to the mean, there is a smaller CV. Therefore, when the channels are more uniform there will be a small CV, which also indicates homogeneity of muscle fibers.

### 2.6. Statistical Analysis

**Figure 4:**
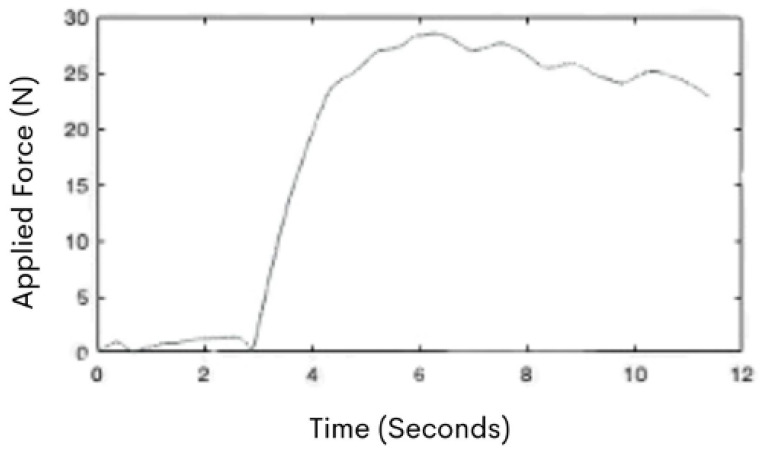
An example of the force curve graph during a participant’s push. The *x*-axis of the graph represents time (s), while the *y*-axis represents force (N).

Data were summarized as mean and SD for each group based on the initiation period of the push, which was determined as 0.5 s after the hand force applied to the cart surpassed 20 N (Figure 4).

Prior to data analysis, Shapiro–Wilks tests were used to ensure the normal distribution of the data. Significant differences were determined using a mixed model repeated measures ANOVA when comparing independent (sex and/or muscle) and dependent (handle design and/or hand force) variables. Bonferroni post-hoc analysis was used when an ANOVA resulted in a *p*-value less than the alpha value, set at 0.05. All statistical tests were performed using RStudio 1.0. 136 (RStudio, Boston, MA, USA).

## 3. Results

### 3.1. Spatial Activity Maps

**Figure 5:**
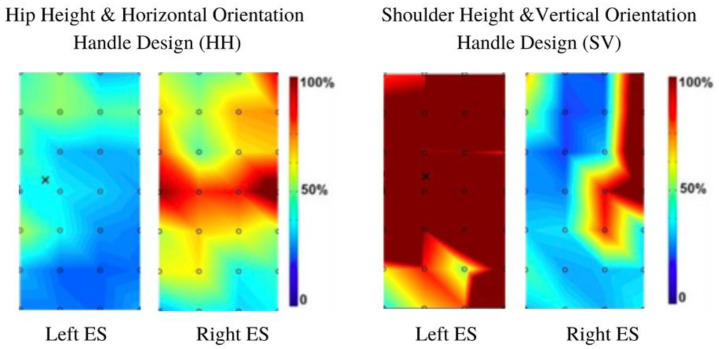
The heat intensity maps from the erector spinae of a female participant when initiating a push.

**Figure 6:**
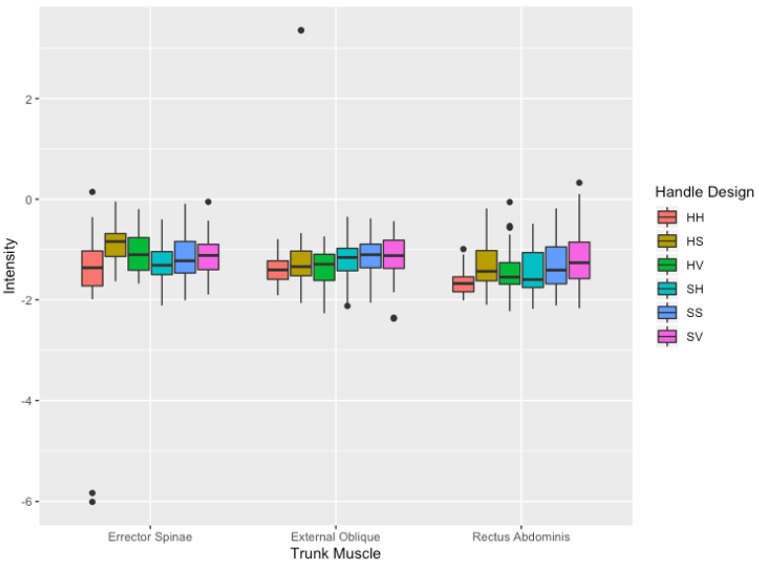
Intensity (mean ± SD) values of the three trunk muscles between handle designs at the initiation of the push. Outliers are noted as black dots. Intensity values are shown as arbitrary units (a.u.).

Color maps were generated for each participant, based on the spatial mean of the RMS of the entire grid area and normalized to each participant MVC. Figure 5 illustrates data from the initiation phase of the pushing task from a sample participant (female) using two different handle designs (each map is scaled to the individual’s MVC). The color deviations in Figure 6 range from dark blue to dark red, indicating low (dark blue) to high levels (dark red) of muscle activation. The left ES showed less overall activity than the right ES with the horizontal handle design at hip height (Figure 5, left pane). When using the vertical orientation handle design at shoulder height, the results for this same participant showed greater activity in the left ES than the right ES. It is important to note that this is an example of one individual.

### 3.2. Spatial Features

The spatial features extracted from the heat intensity maps described muscle activity (intensity, differential intensity, mean RMS) and distribution (entropy and coefficient of variation (CoV)) which were compared across handle designs.

#### 3.2.1. Intensity and Differential Intensity

At the initiation of the push, intensity demonstrated a significant difference for the main effects of handle design (F(5540) = 13.53, *p* > 0.001) as shown in Figure 6. The mean intensity of 4.14 ± 0.84 using the HH design was significantly greater than all other handle designs ((HS: 4.00 ± 0.80; *p* < 0.001); (HV: 4.15 ± 0.62; *p* = 0.002); (SH: 4.10 ± 0.78; *p* = 0.003); (SS: 3.95 ± 0.88; *p* < 0.001); (SV: 3.94 ± 0.82; *p* < 0.001)), regardless of trunk muscle or muscle side. However, the logarithmic equivalent of intensity, differential intensity, resulted in no significant difference across all handle designs and muscles.

#### 3.2.2. Mean RMS

**Figure 7:**
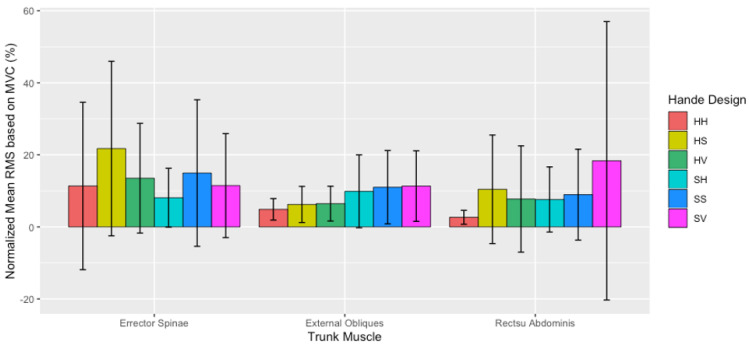
Normalized mean RMS (mean ± SD) based on the percentage (%) of the three trunk muscles between handle designs at the initiation of the push.

The normalized mean RMS showed significant differences for the main effects of handle design (F (5540) = 5.602, *p* < 0.001), as can be seen in Figure 7. The mean RMS was significantly lower using the HH handle design, averaging 6.0 ± 13.0% of the participants MVC, compared to 12.0 ± 17.0% (*p* = 0.027) when using the HS, and 13.0 ± 24.0% (*p* < 0.001) when using the SV handle design, regardless of the trunk muscle or muscle side (Figure 7).

**Figure 8:**
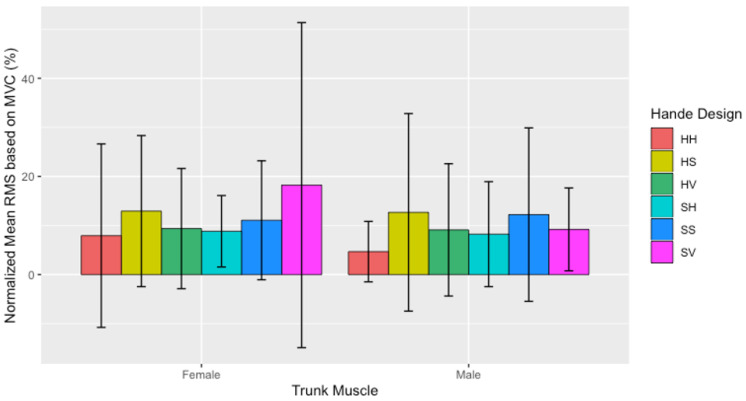
Normalized mean RMS (mean ± SD) based on the percentage (%) across all trunk muscles separated by biological sex between handle designs at the initiation of the push.

There were no significant differences between males and females found in the present study; however, significant differences in the interaction effect involving handle design within biological sex samples were found (F (5540) = 2.313, *p* < 0.001) Particularly, a significant difference in the interaction effect involving handle design and biological sex was found (F (5540) = 2.313, *p* < 0.001) regardless of trunk muscle involved. Within females, initiating a push with the HH handle design averaged significantly less (*p* = 0.035) percentage of their MVC (13.0 ± 2.0%), compared to using the SV handle design (18.0 ± 3.0%). Comparably, within the male sample, initiating a push with the HH handle design averaged significantly less percentage of their MVC (5.0 ± 1.0%) compared to the SS (12.0 ± 2.0%; *p* = 0.041) and HS handle design (13.0 ± 2.0%; *p* = 0.022). Figure 8 illustrates the within-group differences of each group across the six handle designs.

#### 3.2.3. Entropy

**Figure 9:**
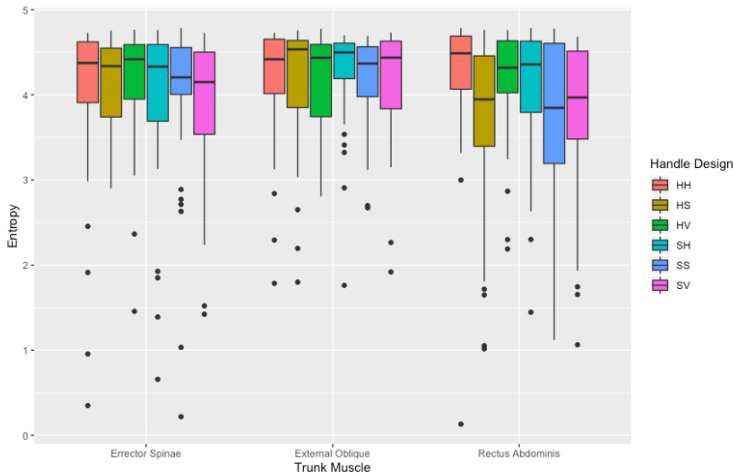
Entropy (a.u) (mean ± SD) values of the three trunk muscles between handle designs at the initiation of the push. Outliers are noted as black dots.

Overall, entropy values exhibited significant differences for the main effects of handle design (F (1540) = 2.521, *p* < 0.001). Further analysis identified that these significant differences between muscle sides were the left-sided erector spinae (ES), external obliques (EO), and rectus abdominis (RA). All left-sided trunk muscles resulted in significantly greater entropy when initiating a push with the HH handle design (ES = 4.27 ± 0.4; EO = 4.26 ± 0.6; RA = 4.25 ± 0.4) than the SS (ES = 3.76 ± 0.9 (*p* = 0.042); EO = 4.05 ± 0.6 (*p* = 0.017); RA = 3.63 ± 1; (*p* = 0.001), and SV handle design (ES = 3.74 ± 0.8 (*p* = 0.020); EO = 4.06 ± 0.7 (*p* = 0.014); RA = 3.66 ± 0.9; (*p* = 0.007)). Mean entropy across all handle designs and muscles are shown in Figure 9.

#### 3.2.4. Coefficient of Variation

**Figure 10:**
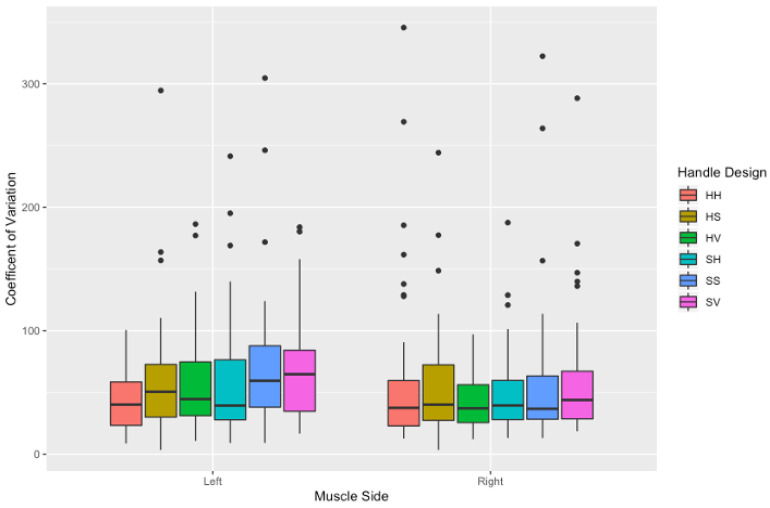
Coefficient of variation (a.u) (mean ± SD) values of the three trunk muscles between handle designs at the initiation of the push. Outliers are noted as black dots.

The coefficient of variation demonstrated a significant difference for an interaction effect of handle design and muscle side (F (5540) = 2.357, *p* < 0.01). In particular, the coefficient of variation of the left-sided erector spinae (ES), external obliques (EO), rectus abdominis (RA) was found to be significantly greater when initiating a push with the SS handle design (ES = 72.62 ± 63.4 (*p* = 0.009); EO = 57.02 ± 28.6 (*p* = 0.012); RA = 77.8 ± 57.4 (*p* = 0.030)) than the HH handle design (ES = 45.09 ± 21; EO = 42.36 ± 26.1; RA = 42.8 ± 24.2), as shown in Figure 10.

## 4. Discussion

Despite the evolution of pushing carts, there have been few advances in industrial cart design. This study used HDEMG to examine spatial features of trunk muscle activity during a simulated industrial cart pushing task. Participants were asked to push a weighted industrial cart with six different handle designs while HDEMG signals were recorded from their trunk muscles. We noted several differences in muscle activity due to handle design which should be considered when designing carts for industrial use.

We found that at the initiation of the push, muscle intensity demonstrated a significant difference for the main effects of handle design. In particular, intensity was higher when using the HH design compared to the other handle designs, regardless of trunk muscle or muscle side. Conversely, mean RMS was significantly lower using the HH handle design compared to the other handle designs, regardless of trunk muscle or muscle side. Contrary to the present study, Lee et al. [2] showed decreased muscle activity and trunk stiffness when participants pushed a 200 kg cart at shoulder height compared with the hip height. Furthermore, Lee et al. [2] considered an identical distance for trials as the present study (5 meters) but instead focused on sudden stops with automated breaking wheels of a 200 kg cart, then the initiation phase of the push. However, Lee et al. [2] used bipolar electrodes over participants’ obliques, rectus abdominis, multifidus, longissimus thoracis pars lumborum, iliocostalis lumborum, iliocostalis thoracis, and longissimus thoracis pars thoracis. In this study, we placed 32-channel electrode grids over the erector spinae, rectus abdominis, and external obliques. Aside from the differences in technology, Lee et al. [2] only examined males in their study, whereas the present study compared an equal number of males and females. It is well accepted that there are anthropometric and muscle strength differences between men and women; however, in the present study no significant findings between sexes with respect to handle design were found. However, it is important to note that in this study, handle heights (hip/shoulder) were adjusted to the 85th percentile of males and females [34]. Lastly, the use of HDEMG allowed us to examine spatial features of the muscle activity, which was not done by Lee et al. [2].

In addition to the more conventional EMG variables, such as mean RMS, the added features associated with HDEMG provided new insight of pushing tasks and muscle response. For instance, entropy and CoV differed between handle designs, offering insight on the spatial differences between each handle design and trunk muscles. Both entropy and CoV have been characterized as indicators of heterogeneity in the spatial distribution of muscle fibers [16,21,22]. Moreover, this clustering of fibers has been suggested to indicate low heterogeneity of muscle fibers [21]. Therefore, the heterogeneity of muscle fibers across a given muscle could aid in examining the level of muscle clustering. In the present study, hip-height handle designs resulted in decreased heterogeneity of the trunk muscles, thus demonstrating a greater proportion of muscle being active. This is consistent with an observation by Watanabe et al. [21], indicating that participants’ trunk muscles recruited larger quantities of motor units to complete a given task, thereby allowing researchers to describe a potential motor unit recruitment pattern. Yet, due to the trunk muscle activity being significantly lower among hip-height handle designs, the difference in entropy instead suggests that, during the initiation of the push, trunk muscles shared the load of the task by recruiting additional muscle fibers and, as a result, had minimal muscle activity during the task.

A limitation of the present study was the choice of limiting the push distance to five meters, as opposed to a further and more realistic distance for workers. Current guidelines are based on pushing a 227 kg cart, 200 times per an 8-hour shift over a maximal distance of 33 meters at a time [23]. Completing a similar protocol to the present study but with a longer push distance would allow for more insight regarding handle design and the additional phases of a pushing task, such as the sustained and ending of a push.

The present study involved young healthy men and women; however, they were inexperienced participants only. Generalization to other populations, specifically those working in industries such as manual material handling, should be made cautiously. Lastly, based on the current study’s findings involving muscle side, future studies should incorporate biomechanical modeling to accurately investigate the impacts of foot placement and its impact on trunk muscles when pushing. Most participants self-selected as right-handed and right leg dominant, suggesting that the right side of the participants’ bodies may overcompensate during the task. This is a concern, as all left-sided trunk muscles had greater coefficient of variation values initiating a push with the SS handle design. This indicated that there were further inconsistent muscle patterns observed, therefore increasing the heterogeneity of muscle fibers in the trunk when using such handle designs. Yet, due to the increased amount of muscle activity observed when using the SS handle design, a similar assumption can be made that such handle design resulted in the ineffective use of the muscle fibers patterns and muscle fiber types.

This study was limited to the examination of spatial muscle activity and did not employ biomechanical measurement techniques such as motion capture, which may have provided further insight regarding the load during the pushing tasks. It has been shown that computational models of spinal loading can help to predict spinal loads during occupational activities, such as pushing [39], and help to assess the risk of work-related low back pain [40]. It would be helpful to include this type of biomechanical modeling in future ergonomic studies along with the spatial activity observations.

Pushing tasks are generally referred to as a full-body task [41,42], yet most research is limited to investigating muscle activity of a single area of the body at a time (e.g., knees, shoulders, and low back) [43]. Although the current study is the first of its kind to explore the trunk using HDEMG during a simulated industrial pushing task, it resembles most previous literature as only investigating a single muscle area. Lee et al. [2], however, compared trunk muscles and low back muscles within the same pushing task. Their findings uncovered an interconnected relationship during a push, indicating coactivation and specific roles involved amongst the muscle groups when pushing. Understanding the interrelationships of muscle groups involved during a pushing task is essential for ensuring a more holistic approach to handle design. Therefore, future research is encouraged to investigate if similar HDEMG muscle activity and patterns are seen among other muscle groups such as the upper back, arms, legs, and thighs.

## 5. Conclusions

The purpose of the present study was to examine the impact of handle design on muscle load during pushing tasks It was found that, with respect to the trunk muscles, the HH handle design resulted in lower muscle activity, and therefore may reduce the physiological strain to the worker. To our knowledge, this study is the first to use HDEMG to examine spatial activity in a simulated industrial cart pushing task and demonstrates its use in other dynamic workplace tasks in ergonomic-based research. The information obtained will help to better inform handle design from the perspective of the trunk and with the influence of multiple design considerations. Furthermore, the use of HDEMG in this study provided greater insight regarding muscle function, promoting its effectiveness in future ergonomic-based research involving dynamic tasks. Although this study was limited to the trunk muscles, future work should examine HDEMG features from the lower legs, forearms, etc., to better understand muscle function during pushing tasks. With future research incorporating additional muscle groups, a more suitable ergonomic handle design could be developed to diminish the risk of injury to workers over time.

## Figures and Tables

**Table 1:** Sample characteristics for all participants (*n* = 20). Standard deviations denoted by SD. Weight is measured in kilograms (kg), height is measured in centimeters (cm), and skinfold measurements in mm.

	Men (*n* = 10)	Women (*n* = 10)	Total (*n* = 20)
Height, mean ± SD (cm)	175.4 ± 6.5	166.4 ± 4.2	170.9 ± 7.1
Weight, mean ± SD (kg)	78.5 ± 8.4	64.3 ± 9.0	71.4 ± 11.2
Age, mean ± SD (years)	24.6 ± 4.7	23.9 ± 4.0	24.3 ± 4.3
Rectus Tissue Skinfold (mm)	3.6 ± 7.3	1.8 ± 3.5	2.7 ± 5.7
Sub Iliac Tissue Skinfold (mm)	3.3 ± 6.8	1.5 ± 3.0	2.4 ± 5.2

## Data Availability

The data presented in this study are available on request from the corresponding author. The data are not publicly available due to privacy issues.
